# Low validity of self-report in identifying recent mental health diagnosis among U.S. service members completing Pre-Deployment Health Assessment (PreDHA) and deployed to Afghanistan, 2007: a retrospective cohort study

**DOI:** 10.1186/1471-2458-9-376

**Published:** 2009-10-08

**Authors:** Remington L Nevin

**Affiliations:** 1 United States Africa Command, Combined Joint Task Force Horn of Africa, Camp Lemonier, Republic of Djibouti

## Abstract

**Background:**

Since 1998, the U.S. Armed Forces has used the mandatory Pre-Deployment Health Assessment (PreDHA) screening questionnaire as a means of assessing the health and suitability of U.S. service members for deployment. Limited data exists to quantify the validity of the self-reported PreDHA. This study was conducted to assess the validity of self-reporting in PreDHA to identify deployed service members who have had a recent mental health disorder diagnosis.

**Methods:**

A retrospective cohort study was conducted on 15,195 U.S. service members deployed in support of combat and reconstruction operations in Afghanistan. The Defense Medical Surveillance System (DMSS), the DoD's longitudinal medical surveillance database, was queried to identify cases among the cohort with a recent diagnosis of a pertinent mental health disorder and to obtain those subjects' responses to the PreDHA.

**Results:**

Of the study cohort, 11,179 (73.6%) subjects had a PreDHA available within the DMSS at the time of analysis. A total of 615 subjects (4.0%) had one or more mental health disorder diagnoses during the pre-deployment period. Out the 615 subjects with diagnosed mental health disorders, 465 had a PreDHA. Among these, only 224, not quite half, answered in the affirmative to the PreDHA question: *"During the past year, have you sought counseling or care for your mental health?"*

**Conclusion:**

This study demonstrates that the self-reported PreDHA has low validity for identifying service members with diagnosed mental health disorders. The development of electronic decision-support systems which automatically screen electronic health records to identify high-risk service members may prove a valuable component of improved pre-deployment screening processes.

## Background

Within the U.S. military, commanders and medical staff have a shared obligation to ensure that service members under their command or care are free of potentially disqualifying or significant mental health disorders that might affect their suitability for and stability during prolonged deployments. Pre-deployment screening programs that identify service members with a recent history of mental health disorder might therefore be a useful adjunct to the existing multilayered process of screening and selection that occurs during accession into the military [1] and during training and service prior to deployment [2].

The Pre-Deployment Health Assessment (PreDHA) screening questionnaire was introduced by the U.S. Department of Defense (DoD) in 1998 as a means of assessing the health and suitability of service members for deployment [3-5]. According to current DoD policies, PreDHAs are to be completed by all service members deploying to land-based operations for 30 days or longer [6], and are to be completed within the 60 days prior to the expected date of deployment [7]. Once completed, PreDHAs are forwarded to the Armed Forces Health Surveillance Center (AFHSC) for inclusion in the Defense Medical Surveillance System (DMSS), the DoD's longitudinal medical surveillance database [8]. The DMSS integrates information on pertinent service member demographics, inpatient and outpatient medical diagnoses at military and civilian medical facilities, and other pertinent health data [5,8], which permits robust epidemiological analyses of health conditions associated with military service.

Since the development of the PreDHA, concerns over the rising mental health consequences of the conflicts in Iraq and Afghanistan have been well described [9-17]. Lowered standards for acceptance of recruits into the military and repeated deployments have combined to significantly increase the prevalence of mental health disorders among U.S. military personnel, including ADHD [18], PTSD [9,14,15,17], anxiety [9], and depression [9,17,19]. As a result, the risk is increased that service members with disqualifying mental health disorders will be inappropriately deployed, and that service members will be prescribed and exposed to medications such as mefloquine (a commonly used anti-malarial), whose contraindications include a history of certain mental health disorders [20].

It has been noted that screening programs are often developed and implemented with minimal evidence as to their efficacy [21]. Between January 2003 and July 2007, over 1,705,787 PreDHA had been administered to deploying U.S. military personnel [5], but to date no published studies have been performed to quantify either the validity or utility of their administration, or to determine how the potential benefits of administering the PreDHA compare to the possible psychological, financial and opportunity costs of implementing the PreDHA screening program [4].

To assess the validity of self-report among service members completing PreDHA, and to quantify the test characteristics of selected questions on the PreDHA for identifying deployed service members with a recent mental health disorder diagnosis, a retrospective cohort study was performed on a large group of U.S. service members deployed to Afghanistan in 2007.

## Methods

### Data Sources, Inclusion and Exclusion Criteria

The U.S. military collects and records pertinent demographic information on service members deploying to combat zones and to other areas of the world in support of contingency operations in a database known as the Defense Theater Accountability System (DTAS). In support of this study, DTAS was queried for a listing of all U.S. military personnel deployed in support of combat and reconstruction operations in Afghanistan as members of the North Atlantic Treaty Organization (NATO) International Security and Assistance Force (ISAF) [22] as of a reference date in early 2007, and this listing was provided to staff at AFHSC for further data queries using the DMSS.

To ensure consistency of demographic data, service members who did not have demographic records available in the DMSS were excluded from analysis. To improve the completeness of data available for analysis, service members with fewer than 30 days of continuous deployment as of the reference date were also excluded. Among remaining service members, the date of the last PreDHA completed and available for analysis within the DMSS was determined, and if there was a record of a PreDHA completed after the DTAS date of deployment, the DTAS deployment date was deemed questionable and the service member was excluded from analysis. All remaining service members comprised the study cohort.

### Demographic and Military Service Data

For each member of the study cohort, the DMSS was queried to determine basic demographic and military service data, including gender, number of prior deployments, and military occupational specialty (combat vs. non-combat). Age was calculated as of the reference date.

### Mental Health History

For each member of the study cohort, the DMSS was queried for evidence of International Classification of Diseases, 9^th ^Edition, Clinical Modification (ICD-9CM) [23] coded diagnoses of pertinent inpatient or outpatient mental health diagnoses, to include major depressive disorder (ICD-9CM 296.2-296.3), adjustment disorder with depressed mood (ICD9-CM 309.0 and 309.28), prolonged depressive reaction (ICD-9CM 309.1), dysthymic disorder (ICD-9CM 300.4), depression (ICD-9CM 311), cyclothymic disorder (ICD-9CM 301.13), generalized anxiety disorder (ICD-9CM 300.02), nonorganic psychoses and schizophrenia (ICD-9CM 298 and 295), bipolar and manic disorders (ICD-9CM 296.0-296.1, 296.3-296.8), obsessive-compulsive disorder (ICD-9CM 300.3), panic disorder, with or without agoraphobia (ICD-9CM 300.01, 300.21), attention-deficit disorders, with or without hyperactivity (ICD-9CM 314.0), dissociative, conversion and factitious disorders (ICD-9CM 300.1), delusional disorders (ICD-9CM 297), and post-traumatic stress disorders (ICD-9CM 309.81). Sub-coded ICD-9CM diagnoses (i.e. those with additional digits of precision) were included. Excluded from analysis were drug and alcohol-related disorders, transient or poorly-defined conditions, phobic disorders, personality disorders, sexual and gender identity disorders, and somatoform diagnoses. A recent mental health disorder diagnosis was defined as one or more pertinent primary or secondary ICD-9CM codes recorded in the full year prior to the deployment date.

### Pre-Deployment Health Assessments

To permit analysis of data from study cohort subjects who might have experienced delays in deployment, all PreDHAs within the 90 days prior to the DTAS deployment date and available in DMSS at the time of analysis were evaluated for pertinent subject and health care provider responses.

**Figure 1:**
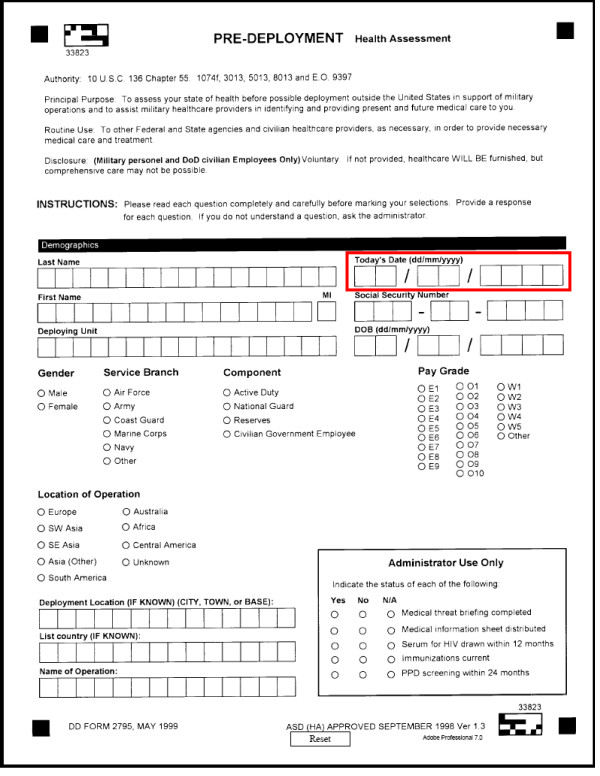
**Pre-Deployment Health Assessment (PreDHA), DD (Defense Department) Form 2795**. page 1. Responses systematically evaluated are outlined in red.

**Figure 2:**
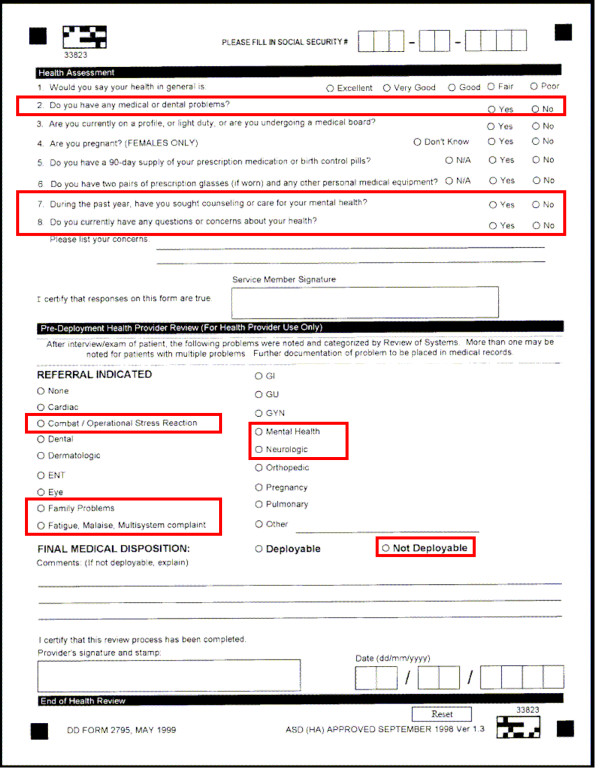
**Pre-Deployment Health Assessment (PreDHA), DD (Defense Department) Form 2795**. page 2. Responses systematically evaluated are outlined in red.

Pertinent subject responses included those to three questions: question #7 *"During the past year, have you sought counseling or care for your mental health?"*; question #2 *"Do you have any medical or dental problems?"*; and question #8 *"Do you currently have any questions or concerns about your health?"*. Pertinent health care provider responses included those to two questions: *"referral indicated" *and *"final medical disposition"*. Responses considered pertinent included a referral for *"combat/operational stress reaction"*, *"family problems"*, *"fatigue, malaise, multisystem complaint"*, *"mental health"*, and *"neurologic" *problems, or a final medical disposition of *"not deployable"*. Pages 1 and 2 of the current two-page PreDHA instrument are enclosed as Figure 1 and Figure 2, respectively; with questions systematically evaluated for this analysis outlined in red.

### Statistical Analysis and Methods

The demographic characteristics of the study cohort and those with a pre-deployment mental health disorder diagnosis were compared by Chi-square test across demographic strata, and odds ratios (OR) and 95% confidence intervals (CI) were calculated for dichotomous strata. Numbers of subjects with recent mental health disorder diagnosis by ICD-9CM coded diagnosis category were determined. Numbers of subjects in the study cohort and with a recent mental health disorder diagnosis with available PreDHAs within 90 days prior to the DTAS deployment date were compared by Chi-square test. Among those with a recent mental health disorder diagnosis, the OR for the presence of multiple available PreDHAs within 90 days of deployment was assessed.

To evaluate the test characteristics of the PreDHA, subjects with one or more PreDHA completed in the 90 days prior to deployment were stratified by presence of recent mental health disorder diagnosis, and the presence of pertinent responses to questions from the most recent PreDHA were assessed to calculate the odds ratio (OR) and "number needed to screen" (NNS) for each. In contrast to other uses of this statistic [21], for this study the NNS was defined as the number of subjects needed to be screened with the PreDHA in the 90 days prior to deployment to obtain a positive response to one or more pertinent questions on the most recent PreDHA in the presence of a recent mental health disorder diagnosis. Additional odds ratios were assessed comparing presence of referral or non-deployable status and positive responses to subject-reported data. Data manipulation was performed using SAS 9.1 [24]. Odds ratios and 95% confidence intervals were obtained via the Cornfield approximation [25] using Stata 8.0.

### Ethical Considerations

This analysis was conducted by a duly constituted public health authority to evaluate the effectiveness of military medical screening programs and policies. Data were requested from AFHSC under military command approval, with analysis performed consistent with Department of Defense Directives (DoDD) 6590.2 and DoDD 6490.02E and as non-research exempt from Institutional Review Board (IRB) requirements in accordance with United States Code of Federal Regulations (CFR) section 38 CFR 16 ("The Common Rule"). Identified health data were securely transmitted, processed and retained on data systems compliant with DoD information assurance policies.

## Results

### Subject Demographics and Mental Health History

A list of 18,733 U.S. service members was provided to AFHSC, and after application of exclusion criteria, the final study cohort comprised 15,195 subjects. A total of 615 subjects (4.0%) had one or more mental health disorder diagnoses in the year prior to deployment. Females had over twice the odds of having a recent mental health disorder diagnosis (OR = 2.26, 95% CI 1.82-2.80) as males (P < 0.001, χ^2 ^= 58.09). Subjects with one or more prior deployments had significantly greater odds of having a recent mental health disorder diagnosis (OR = 1.44, 95% CI 1.22-1.70) as those without (P < 0.001, χ^2 ^= 19.18). Those in non-combat occupations had significantly greater odds of having a recent mental health disorder diagnosis (OR = 1.33, 95% CI 1.09-1.62) as those in combat occupations (P = 0.006, χ^2 ^= 7.69). There were no significant differences between the study cohort and those with a recent mental health disorder diagnosis by age category (P = 0.273, χ^2 ^= 5.14). Demographic data is shown as Table 1.

**Table 1:** Demographics of the study cohort^1 ^and those with a recent mental health disorder diagnosis^2^

	**Study cohort**		**Subjects with a recent mental health disorder diagnosis**	
	
	**Number**	**(Percentage)**	**Number**	**(Percentage)**
**Total**	15195	(100)	615	(100)
**Gender**				
Male	13816	(90.9)	506	(82.3)
Female	1379	(9.1)	109	(17.7)
**Prior deployments**				
0	10180	(67.0)	362	(58.9)
1+	5015	(33.0)	253	(41.1)
**Military occupation**				
Combat	3810	(25.1)	125	(20.3)
Non-combat	11385	(74.9)	490	(79.7)
**Age**				
18-19	503	(3.3)	22	(3.6)
20-29	9558	(62.9)	396	(64.4)
30-39	3737	(24.6)	151	(24.6)
40-49	1255	(8.3)	45	(7.3)
50+	142	(0.9)	1	(0.2)

Note: Significant difference by gender (P < 0.001), prior deployment (P < 0.001), and military occupation (P = 0.006) by Chi-square.

1. Demographic characteristics as of the deployment date.

2. Record of one or more pertinent ICD-9CM diagnosis codes in the full year prior to date of deployment.

Of the 615 deployed subjects with a recent mental health disorder diagnosis, 17 (2.3%) had one or more inpatient diagnoses, while 8 (1.3%) had one or more primary inpatient diagnoses. All 615 had one or more outpatient diagnoses, while 531 (86.3%) had one or more primary outpatient diagnoses. The number of subjects with specific diagnoses, by ICD-9CM codes and type of diagnosis are shown as Table 2.

**Table 2:** Numbers of subjects with a recent mental health disorder diagnosis^1^, by ICD-9-CM diagnosis code and encounter type

		**Number with any diagnosis**				
		
**Diagnosis**	**ICD-9CM diagnosis code**	**Any**	**Inpatient**		**Outpatient**	
			
			**Primary**	**Any**	**Primary**	**Any**
Major depressive disorder	296.2-296.3	61	3	3	52	61
Adjustment disorder with depressed mood	309.0, 309.28	265	4	4	232	265
Prolonged depressive reaction	309.1	4	0	0	4	4
Dysthymic disorder	300.4	46	0	1	35	45
Depression	311	190	0	5	140	190
Cyclothymic disorder	301.13	2	0	0	2	2
Generalized anxiety disorder	300.02	19	0	0	15	19
Nonorganic psychoses and schizophrenia	295, 298	5	0	1	4	4
Bipolar and manic disorders	296.0-296.1, 296.4-296.8	14	0	0	12	14
Obsessive-compulsive disorder	300.3	8	0	0	7	8
Panic disorder, with or without agoraphobia	300.01, 300.21	19	0	0	13	19
Attention-deficit disorders, with or without hyperactivity	314.0	101	0	0	93	101
Dissociative, conversion and factitious disorders	300.1	7	0	0	6	7
Delusional disorders	297	1	0	0	1	1
Post-traumatic stress disorder	309.81	74	1	5	58	72

**Any**		**615**	**8**	**17**	**531**	**615**

1. Record of one or more pertinent ICD-9CM diagnosis codes in the full year prior to date of deployment.

Of the 8 deployed subjects with one or more primary inpatient diagnoses, 3 were diagnosed with major depressive disorders, 4 with adjustment disorder with depressed mood, and 1 with post-traumatic stress disorder. Of the 8, all (100%) had no prior deployments, and 5 (62.5%) were female.

### Pre-Deployment Health Assessments

Of the study cohort 11,179 (73.6%) subjects had at least one PreDHA available for analysis that was completed within the 90 days prior to deployment. Of these, a total of 1,633 (14.6%) subjects completed multiple PreDHAs during this period. The odds of a recent mental health disorder diagnosis were greater among those with one or more PreDHA available as compared to those without a PreDHA (OR = 1.12, 95% CI 0.93 - 1.35), although this difference was not statistically significant (P = 0.242, χ^2 ^= 1.37). Among subjects with at least one PreDHA available for analysis and completed within the 90 days prior to deployment, those with a recent mental health disorder diagnosis also had greater odds of completing multiple PreDHA in the 90 days prior to deployment (OR = 1.19, 95% CI 0.93 - 1.52) than of completing only one, but this also did not reach statistical significance (P = 0.177, χ^2 ^= 1.83).

Of the 8 deployed subjects with one or more primary inpatient diagnoses, 7 had PreDHAs available, of which 6 (85.7%) answered in the affirmative to question #7, 2 (28.6%) in the affirmative to question #2, and none (0%) in the affirmative to question #8.

### PreDHA Responses and Test Characteristics

Among the 11,179 with one or more PreDHA completed in the 90 days prior to deployment, 465 (4.2%) had one or more recent mental health disorder diagnoses. Of the 465, 224 (48.2%) responded in the affirmative to question #7.

Those with a recent mental health disorder diagnosis were significantly more likely to answer question #7 in the affirmative (OR = 41.8, 95% CI 33.4 - 52.3) than those without (P < 0.001, χ^2 ^= 2404). The odds ratio for question #2 was considerably lower (OR = 2.88, 95% CI 2.30 - 3.60), as was that for question #8 (OR = 2.28, 95% CI 1.56 - 3.35), but both were statistically significant, (P < 0.001, χ^2 ^= 93.1) and (P < 0.001, χ^2 ^= 18.5) respectively. The OR and NNS for each pertinent PreDHA question are tabulated as Table 3.

**Table 3:** Test characteristics of PreDHA questions in identifying a recent mental health disorder diagnosis^1 ^among subjects with available PreDHA^2^

	**Odds Ratio**	**Number Needed to Screen**
**Subject self-reported data**		
"During the past year, have you sought counseling or care for your mental health?"	41.8	49.9
"Do you have any medical or dental problems?"	2.9	102.6
"Do you currently have any questions or concerns about your health?"	2.3	372.6
Positive response to any of the above	10.1	40.5
**Provider-reported data**		
Referral indicated	17.1	859.9
Medical disposition "not deployable"	2.6	532.3
Positive response on one or more of the above	3.5	372.6
**Any of the above**		
One or more of the above	9.5	39.6

1. Record of one or more pertinent ICD-9CM diagnosis codes in the full year prior to date of deployment.

2. Within 90 days prior to DTAS deployment date.

### Referrals

Among all subjects completing one or more PreDHA in the 90 days prior to deployment, a total of 31 subjects (0.3%) received a pertinent referral. Among the 19 (61.2%) referred subjects indicating a positive response to any of questions #7, #2 or #8, the odds of referral were over 9 times higher (OR = 9.34, 95% CI 4.60 - 19.0) than among those without any positive response (P < 0.001, χ^2 ^= 54.2). Those endorsing question #7 in the affirmative were considerably more likely to be referred (OR = 33.8, 95% CI 16.7 - 68.4) as compared to those who did not (P < 0.001, χ^2 ^= 231); as were those endorsing question #2 (OR = 4.89, 95% CI 2.37 - 10.1) as compared to those who did not (P < 0.001, χ^2 ^= 21.7). Those endorsing question #8 in the affirmative were also more likely to be referred (OR = 3.40, 95% CI 1.09 - 10.6) than those who did not (P = 0.033, χ^2 ^= 4.54). Among those who specifically answered question #7 in the negative, those with a mental health disorder diagnosis were significantly more likely to receive a referral (OR = 13.2, 95% CI 3.90 - 44.8) than those without (P < 0.001, χ^2 ^= 25.7).

### Non-Deployable Final Medical Disposition

Among those who answered question #7 in the negative, those with a recent mental health disorder diagnosis were more likely to receive a final medical disposition of *"not deployable" *than those without (OR = 1.68, 95% CI 0.80 - 3.56), but this difference was not statistically significant (P = 0.178, χ^2 ^= 1.82).

Among the study cohort, there were a total of 210 subjects who were deployed when their most recent PreDHA was annotated by the health care provider to say their final medical disposition was *"not deployable"*. Of these, 21 (10.0%) had a recent mental health disorder diagnosis; none had an inpatient diagnosis, and 17 (81.0%) had one or more primary outpatient diagnoses. Of the 21, 4 (19%) had 2 prior deployments, 7 (33.3%) had 1 prior deployment, and the remainder had none. Of the 21, 5 (23.8%) were female. Of the 21, 14 (66.7%) answered in the affirmative to question 7, 12 (57.1%) to question 2, and 3 (14.3%) in the affirmative to question 8.

## Discussion

This is the first study to assess the sensitivity and other test characteristics of responses to PreDHA questions for identifying a recent mental health disorder diagnosis among deployed U.S. service members. This study found a prevalence of recent mental health disorder diagnosis of 4.2% among those with one or more PreDHA completed in the 90 days prior to deployment and available for analysis in DMSS. Although this study found that subjects with a recent mental health disorder diagnosis were significantly more likely to answer pertinent questions on the PreDHA in the affirmative, more than half of such subjects provided no indication of their diagnosis on their most recent PreDHA.

This study found that the sensitivity of the question *"during the past year, have you sought counseling or care for your mental health?" *in identifying a recent mental health disorder diagnosis was only 48.2%. Subjects with a recent mental health disorder diagnosis were over 41 times as likely to answer this question in the affirmative as those without, but this study also found that almost 50 subjects needed to be screened with PreDHA to identify one subject with a recent mental health disorder diagnosis who indicated such a response. Including affirmative responses to additional relevant questions, including those inquiring about current health concerns or problems did not appreciably increase the sensitivity of self-reported data. Furthermore, although subjects with a recent mental health disorder diagnosis were over twice as likely to endorse current medical or dental problems (OR = 2.88), or to have questions or concerns about their health as those without (OR = 2.28), only 23.4% of those with a mental health disorder diagnosis endorsed current medical or dental problems, and only 6.5% endorsed any current questions or concerns about their health.

These findings fit with those of other researchers, who have noted among deploying and redeploying U.S. service members perceptions of significant stigma associated with mental health conditions [9-11], and a tendency among U.S. service members towards underreporting as well as a preference towards declining treatment. A major study of U.S. service members preparing for deployment to Iraq found among those who screened positive for major depression, generalized anxiety, or PTSD, 86% acknowledged a problem, but only 40% were interested in receiving help [9]. Among a larger sample of service members screening positive, a majority indicated perceived barriers to receiving mental health care, including concerns that others in their unit would lose confidence in them, view them as being weak, or blame them for their mental health problems [9]. The expression of these concerns might be expected to increase in the context of mass-screening programs such as PreDHA which are often conducted at unit-level.

Perhaps not surprisingly, therefore, this study found a very low rate of pertinent health care provider referrals. Less than one-third of 1% of all subjects with an available PreDHA (0.3%) received such a referral. Almost 860 subjects had to be screened with PreDHA to result in a single pertinent referral for a subject with a recent mental health disorder diagnosis. Of subjects receiving such a referral, approximately 6 in 10 (61.2%) had answered in the affirmative to one or more pertinent question on their most recent PreDHA, and those indicating a positive response to such questions were over nine times (OR = 9.34) as likely to be referred as those who did not. According to current guidance [7], upon completion by the service member, responses to PreDHA are to be immediately reviewed by a *"medic, nurse, medical technician or corpsman"*, and any positive responses to (among others) questions 7, 2 and 8 *"requires referral to a trained health care provider (physician, physician assistant, nurse practitioner, advanced practice nurse, independent duty corpsman, independent duty medical technician, or Special Forces medical sergeant)" *[7]. Existing DoD guidance is not clear as to whether this referral requires appropriate additional annotation by the trained health care provider on the PreDHA, or merely requires the PreDHA to be reviewed and certified by such a provider.

Of note in this analysis, subjects who had a recent mental health disorder diagnosis but denied having such a history were not statistically more likely to be identified by the provider as *"not deployable" *than those without such a history, which suggests that these subjects might have been able to successfully conceal or minimize a potentially disqualifying condition. Whether the 21 *"not deployable" *subjects with a mental health disorder diagnosis were appropriately deployed in accordance with current DoD guidance and regulations is not clear by this study's methodology. Existing DoD instructions [7] describe only the manner in which PreDHAs are to be completed, but do not provide guidance as to what patterns of subject-provided responses constitutes a disqualification for deployment; nor do they formalize a mechanism for ensuring that service members flagged by the provider as *"not deployable"*, in fact, are not deployed. A recent policy memorandum drafted in response to Congressional concerns [26]*"provides guidance on deployment... for military personnel who experience psychiatric disorders" *and states that *"diagnosed conditions that are not amenable or anticipated not amenable to treatment and restoration to full functioning within one year of treatment should generally be considered unfitting...." *The memorandum specifically states that *"psychotic and bipolar disorders are considered disqualifying for deployment"*. This study found evidence of 5 subjects with diagnoses of psychoses and 14 subjects with diagnoses of bipolar and manic disorder. Whether these subjects were appropriately deployed is also not clear by this study's methodology.

This study has a number of important limitations that require the results to be interpreted in context. Most significantly, this study included only service members who were identified on official rosters as being deployed; not the larger cohort of service members who were administered PreDHA in anticipation of deployment. Limitations in data available within the DMSS precluded accurately identifying this cohort; future studies using alternate methodologies might assess the number of service members who may have been identified as *"not deployable" *and who did not subsequently deploy.

This study, as with previous research on deployment-related mental health issues [12,27], relied upon medical surveillance data in the DMSS to identify the presence of pertinent mental health disorder diagnoses. The strengths and limitations of this data have been previously described [12], although research has demonstrated good correlation between ICD-9CM coded mental health disorder diagnoses and evidence of psychoactive pharmacotherapy [28], confirming the utility of surveillance databases in identifying functional psychiatric morbidity.

This study examined only records of ICD-9CM diagnostic codes corresponding to pertinent mental health disorders, and did not assess ICD-9CM "V-coded" visits for counseling not associated with a formal diagnosis [29], nor visits to social workers, chaplains, and other counselors practicing outside of administrative systems whose data is captured by the DMSS. Including such data as evidence of a recent mental health disorder diagnosis might have improved the validity and test characteristics of the PreDHA. For example, making the assumption that all subjects without evidence of diagnosis who had answered question #7 in the affirmative, in fact, had received some form of counseling or care for a mental health disorder, independent of documented diagnosis, would have increased the sensitivity of the question, as written, to 65.6% and decreased the NNS to 24.5 (data not shown).

This study included ADHD in the definition of a mental health disorder diagnosis. Although a remote history of ADHD may not always be considered problematic, in U.S. military settings a recent active diagnosis of ADHD remains highly relevant. A recent study of health care operations in Iraq [30] confirms that *" [d]eployment of soldiers with chronic mental health disorders such as anxiety, attention deficit disorder, and depression is problematic*..." Furthermore, the previously mentioned policy memorandum [26] states "*[p]sychotropics clinically and operationally problematic during deployments include... stimulants*", which are commonly prescribed to treat ADHD. In the present analysis, of the 615 subjects with a recent mental health disorder diagnosis, 101 subjects (16.4%) were identified with a recent diagnosis of attention-deficit disorders, with or without hyperactivity. Of these, the vast majority (92%) received primary outpatient diagnosis, highly suggestive of active disorder. Furthermore, there is a high prevalence of treated attention-deficit disorder conditions among deployed personnel as suggested by a recent published study [20], in which 78 of 11,725 deployed subjects (0.67%) had been prescribed a pharmacologic treatment for ADHD prior to deployment; highly comparable to the 101 of 15,195 (0.66%) diagnosed with an attention-deficit spectrum diagnosis in this present analysis.

As this analysis examined electronic medical records only for evidence of a recent diagnosis, remote histories not requiring continued treatment would not necessarily have been identified. Additionally, while ADHD is recognized professionally as a mental health diagnosis, service members who consider their condition as other than a *"mental health issue or problem" *may have responded in the negative to the question on the PreDHA despite recent diagnosis. Future analyses should be performed to address to what extent perception of this condition, and other conditions, could have influenced the results of this study.

This study examined only PreDHA data successfully integrated and available for analysis in the DMSS. Of the study cohort, a total of 4,016 subjects (26.4%) had no PreDHA available within the 90 days prior to deployment. Of the subjects without an available PreDHA, 150 (3.7%) had a mental health diagnosis. The methodology of this study was unable to determine whether a PreDHA was actually administered to these subjects. Although completion of the PreDHA is mandatory, no formal system exists to validate the correct transmission of PreDHA data from military service-specific sources to AFHSC, nor to provide timely confirmation to the service member, his or her commander, or his or her health care provider of successful integration of the service member's data into the DMSS prior to deployment.

Independent of these limitations, this study demonstrates that the subject-reported data collected during the PreDHA process is of questionable validity. Existing policy [26] states that *"it is the responsibility of the Service member to report past or current physical or mental health conditions or concerns and associated treatments"*. The results of this study provide strong evidence that relying on self-report alone may be insufficient policy for screening for disqualifying or significant mental health conditions. These results support the recent conclusions of the Department of Defense Task Force on Mental Health, a special body established at the direction of Congress to *"examine matters relating to mental health and the Armed Forces"*. In the Task Force's final report, it was noted that its members *"were told on multiple site visits that the validity of the Pre-Deployment Health Assessment suffers because service members underreport their mental health concerns..." *[31]

In the policy memorandum announcing the introduction of the PreDHA in 1998 [3], it was noted that the objective of the assessments was merely to provide *"quick confirmation and documentation of a service member's health readiness for deployment or redeployment and to determine if there is a need for a clinician's evaluation before deployment or redeployment. Future revisions of deployment health assessments shall require pilot testing and question validation before being put into use" *[3]. Furthermore, this memorandum stated that *"deployment-related mental health screening will be addressed in a separate policy memorandum"*. Since this original announcement released over ten years ago, no such policy memorandum has been issued, and no formal validation of the PreDHA has been published or undertaken.

## Conclusion

This study demonstrates that self-report by PreDHA has low validity in identifying members of the U.S. military with a recent mental health disorder diagnosis. In light of this study's findings and in consonance with prior recommendations to improve the quality of mental health care provided to U.S. military service members, consideration should be given to complementing the PreDHA with a validated mental health screening instrument of higher sensitivity [32]. The development of electronic decision-support systems which automatically screen electronic health records, including ICD-9CM diagnosis and pharmacy data, to identify high-risk service members may prove a valuable component of improved pre-deployment processing [33]. Such systems have been proposed previously as a means of improving the safety of prescribing and dispensing of mefloquine, which shares with pre-deployment screening the requirement to accurately identify a history of recent mental health diagnosis [20].

## List of Abbreviations

ADHD: Attention-Deficit/Hyperactivity Disorder; AFHSC: Armed Forces Health Surveillance Center; CFR: Code of Federal Regulations; CI: Confidence Interval; DoD: Department of Defense; DoDD: Department of Defense Directive; DMSS: Defense Medical Surveillance System; DTAS: Defense Theater Accountability System; ICD-9CM: International Classification of Diseases; 9^th ^Edition; Clinical Modification; IRB: Institutional Review Board; ISAF: International Security and Assistance Force; NATO: North Atlantic Treaty Organization; NNS: Number Needed to Screen; NPV: Negative Predictive Value; OR: Odds Ratio; PreDHA: Pre-Deployment Health Assessment; PPV: Positive Predictive Value; PTSD: Post-Traumatic Stress Disorder; U.S.: United States.

## Competing interests

The author declares that they have no competing interests.

## Author's Information

The author was deployed to Afghanistan as a U.S. Army Preventive Medicine physician in support of the ISAF combat and reconstruction mission from January 2007 through October 2007.

## Pre-publication history

The pre-publication history for this paper can be accessed here:


